# Resistance-Nodulation-Division Efflux Pump, LexABC, Contributes to Self-Resistance of the Phenazine Di-*N*-Oxide Natural Product Myxin in *Lysobacter antibioticus*

**DOI:** 10.3389/fmicb.2021.618513

**Published:** 2021-02-17

**Authors:** Yangyang Zhao, Jiayu Liu, Tianping Jiang, Rongxian Hou, Gaoge Xu, Huiyong Xu, Fengquan Liu

**Affiliations:** ^1^Institute of Plant Protection, Jiangsu Academy of Agricultural Sciences, Jiangsu Key Laboratory for Food Quality and Safety-State Key Laboratory Cultivation Base of Ministry of Science and Technology, Nanjing, China; ^2^College of Plant Protection (Key Laboratory of Integrated Management of Crop Diseases and Pests), Nanjing Agricultural University, Nanjing, China; ^3^Institute of Life Sciences, Jiangsu University, Zhenjiang, China

**Keywords:** phenazine, RND pump, antibiotic resistance, LysR-type regulator, *Lysobacter antibioticus*

## Abstract

Antibiotic-producing microorganisms have developed several self-resistance mechanisms to protect them from autotoxicity. Transporters belonging to the resistance- nodulation-division (RND) superfamily commonly confer multidrug resistance in Gram-negative bacteria. Phenazines are heterocyclic, nitrogen-containing and redox-active compounds that exhibit diverse activities. We previously identified six phenazines from *Lysobacter antibioticus* OH13, a soil bacterium emerging as a potential biocontrol agent. Among these phenazines, myxin, a di-*N*-oxide phenazine, exhibited potent activity against a variety of microorganisms. In this study, we identified a novel RND efflux pump gene cluster, designated *lexABC*, which is located far away in the genome from the myxin biosynthesis gene cluster. We found a putative LysR-type transcriptional regulator encoding gene *lexR*, which was adjacent to *lexABC*. Deletion of *lexABC or lexR* gene resulted in significant increasing susceptibility of strains to myxin and loss of myxin production. The results demonstrated that LexABC pump conferred resistance against myxin. The myxin produced at lower concentrations in these mutants was derivatized by deoxidation and *O*-methylation. Furthermore, we found that the abolishment of myxin with deletion of *LaPhzB*, which is an essential gene in myxin biosynthesis, resulted in significant downregulation of the *lexABC.* However, exogenous supplementation with myxin to *LaPhzB* mutant could efficiently induce the expression of *lexABC* genes. Moreover, *lexR* mutation also led to decreased expression of *lexABC*, which indicates that LexR potentially positively modulated the expression of *lexABC*. Our findings reveal a resistance mechanism against myxin of *L. antibioticus*, which coordinates regulatory pathways to protect itself from autotoxicity.

## Introduction

Phenazines are a large class of heterocyclic, nitrogen-containing and redox-active natural products exhibiting a wide range of biological activities including antibiotic, antitumor, biofilm modulation and so on. Since the first blue phenazine pyocyanin (PYO) was identified, over 180 natural phenazines have been isolated and more than 6,000 phenazines have been chemically synthesized. Natural phenazines have been identified as secondary metabolites primarily from *Pseudomonas* and *Streptomyces* (Laursen and Nielsen, 2004; Cimmino et al., 2012; Guttenberger et al., 2017).

**FIGURE 1:**
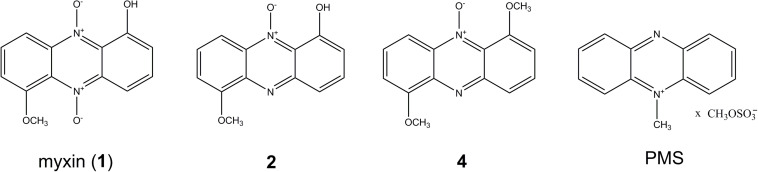
Structures of phenazines from *L. antibioticus* OH13 and PMS (phenazine methosulfate).

*Lysobacter*, a genus of Gram-negative bacteria, is emerging as a novel source of biocontrol agents against phytopathogenic microorganisms because they have the ability to produce abundant active lytic enzymes and secondary metabolites (Xie et al., 2012; Panthee et al., 2016; Puopolo et al., 2018). We previously isolated six phenazines from *Lysobacter antibioticus* OH13, of which myxin (Figure 1), an *N*-oxide phenazine, drew much attention due to its potent activity against microbes (Weigele et al., 1970; Zhao et al., 2016) and human cancer cells (Viktorsson et al., 2017).

Our previous work revealed that myxin was derived from phenazine 1,6-dicarboxylic acid (PDC) and was biosynthesized by a *LaPhz* gene cluster including six core genes, *LaPhzB-G*, and four modification genes, *LaPhzNO1*, *LaPhzS*, *LaPhzM*, and *LaPhzX*. We first experimentally characterized LaPhzNO1, homologous to Baeyer-Villiger flavoproteins catalyzing phenazine *N*-oxidation (Zhao et al., 2016). The unique feature of myxin is that it not only belongs to the class of phenazine compounds but also to a class known as heterocyclic *N*-oxides with bioreductively activated, hypoxia-selective DNA-damaging properties (Mfuh and Larionov, 2015). Early studies showed that myxin interacted with DNA through intercalation and inhibited DNA template-controlled RNA synthesis (Hollstein and Van Gemert, 1971; Hollstein and Butler, 1972). More recent findings indicated that myxin caused bioreductively activated, radical-mediated DNA strand cleavage under both aerobic and anaerobic conditions (Chowdhury et al., 2012). The unique mode of action and strong biological activities of myxin make it an attractive target of study and development.

Antibiotic-producing microorganisms have evolved several self-resistance mechanisms to prevent autotoxicity, such as antibiotic efflux, antibiotic modification, self-sacrifice, target repair or protection (Ogawara, 2019). One of the self-resistance mechanisms is that efflux pumps transport toxic compounds outside of the cells (Yan et al., 2020). Efflux pumps have been classified into five major groups based on their protein sequences, which are RND (resistance-nodulation-cell division), MFS (major facilitator superfamily), MATE (multidrug and toxic compound extrusion), SMR (small multidrug resistance), and ABC (ATP-binding cassette) superfamilies or families (Delmar et al., 2014).

RND efflux pumps play important roles in multidrug resistance (MDR) due to their broad-spectrum substrate profile in Gram-negative bacteria. The RND efflux systems are composed of a tripartite complex: an inner membrane protein (IMP), an outer membrane protein (OMP), and a peri-plasmic membrane fusion protein (MFP) that links the IMP and OMP (Li et al., 2015; Venter et al., 2015). The typical RND efflux pump systems, such as the AcrAB-TolC pump of *Escherichia coli* and the Mex pumps in *Pseudomonas aeruginosa*, have been intensively studied (Peterson and Kaur, 2018). The AcrAB-TolC pump has an extremely wide range of substrates and can transport harmful molecules to the extracellular medium (Li et al., 2015). Recently, it was revealed that the AcrAB-TolC pump is essential in tetracycline resistance acquisition by plasmid transfer (Nolivos et al., 2019). RND pumps in *P. aeruginosa* including MexAB-OprM, MexCD-OprJ, MexEF-OprN, MexXY, and MexGHI-OpmD are significant determinants of multidrug resistance against various antibiotic families such as β-lactams, aminoglycosides, fluoroquinolones and phenazines (Li et al., 2015; Sakhtaha et al., 2016). Multiple regulatory components modulate RND pumps, for example, MexAB-OprM is regulated by MexR (Evans et al., 2001), NalD (Morita et al., 2006), CpxR (Tian et al., 2016), PA3898, and PA2100 (Heacock-Kang et al., 2018). LysR-type transcriptional regulator, MexT (Fargier et al., 2012), and AdeL (Coyne et al., 2010) involve in activating the expression of MexEF-OprN and AdeFGH efflux pump, respectively.

We found that myxin showed highly effective inhibition against many bacteria, such as methicillin-resistant *Staphylococcus aureus* (MRSA, MIC 0.05 μg/mL) (Zhao et al., 2016), *Xanthomonas oryzae* pv. *oryzae* (MIC 2 μg/mL), and *Xanthomonas oryzae* pv. *oryzicola* (MIC 2 μg/mL). The antibiotic properties of myxin have been the subject of intense research efforts, but little is known about their effect on the producing organisms, and moreover, how *L. antibioticus* itself responds to and survives in the presence of myxin.

Here, we identified a gene cluster on the chromosome of *L. antibioticus* OH13, *lexABC*, encoding an RND family multidrug efflux pump that confers resistance to myxin and phenazine methosulfate (PMS) (Figure 1). We discovered that the expression of *lexABC* was promoted by myxin and positively regulated by a LysR-type transcriptional regulator, indicating that the LexABC pump could coordinate with myxin production to protect *L. antibioticus* OH13 from autotoxicity.

## Results

### The LexABC Efflux System and LexR Regulator Are Involved in Self-Resistance Against Myxin

**FIGURE 2:**
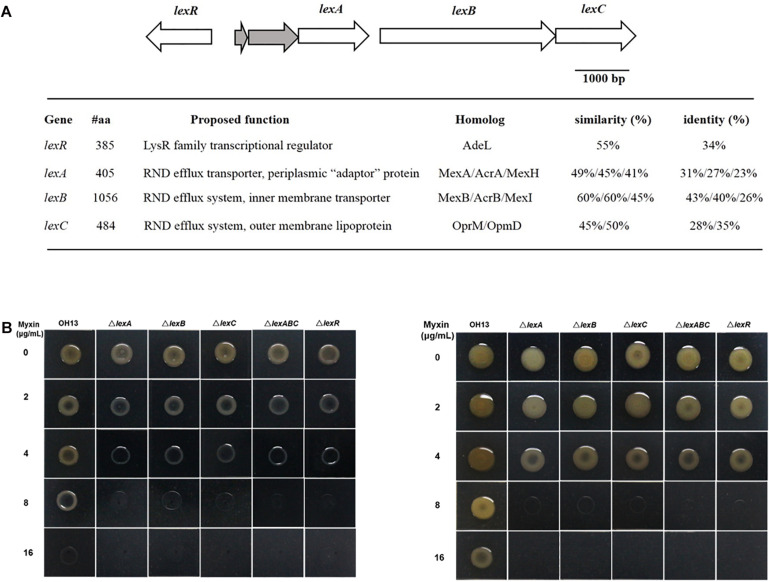
Schematic representation of *lex* genes and susceptibility of different strains to myxin. **(A)** Organization of *lexA*, *lexB*, *lexC*, *lexR* in *L. antibioticus* OH13 and their predicted functions. **(B)** Growth of *L. antibioticus* OH13 and its derivatives on media containing different concentrations of myxin. Left, cultured for 24 h. Right, cultured for 48 h. Images are representative of three independent experiments.

Bioinformatics analysis of *L. antibioticus* OH13 genomic sequence revealed three genes encoding RND efflux pump proteins, which were similar to MexAB-OprM and MexGHI-OpmD from *P. aeruginosa*, located distant from the phenazine biosynthetic gene cluster. We proposed naming these three genes *lexA, lexB*, and *lexC* (NCBI accession no. MW029615, MW029616, MW029617, respectively) for *Lysobacter* phenazine efflux system proteins. LexA had 49%/45%/41% sequence similarity to MexA/AcrA/MexH, and LexB shared 60%/60%/45% similarity with MexB/AcrB/MexI, respectively, whereas LexC was homologous to OprM and OpmD (45 and 50% similarity) but was not a putative TolC-like protein (Figure 2A and Supplementary Figure S1). Moreover, a putative gene encoding LysR family transcriptional regulator, *lexR* (NCBI accession no. MW029618) was found at the upstream of *lexABC* locus. LexR shared 55% similarity with AdeL which was a LysR family transcriptional regulator (Figure 2A and Supplementary Figure S1). To elucidate the role of the LexABC system and assumed regulator LexR in myxin resistance, mutants lacking *lexA*, *lexB*, *lexC*, *lexABC*, and *lexR* (Δ*lexA*, Δ*lexB*, Δ*lexC*, Δ*lexABC*, and Δ*lexR*) were constructed. Next, we tested the contribution of these genes in myxin resistance by growing various mutants on NA medium containing different concentrations of this compound (Figure 2B and Table 1). After culturing for 24 h, the mutants showed severe growth defects on medium with 4, 8, and 16 μg/mL myxin compared to the wild type (Figure 2B and Table 1). At lower myxin concentrations (2 and 4 μg/mL), the growth of the mutants caught up after 48 h cultivation. However, at higher myxin concentrations (8 and 16 μg/mL), the growth of these mutants was completely stopped (Figure 2B and Table 1). And the complemented strains of *lexA*, *lexB*, *lexABC* restored their resistance against myxin to the level of the wild type (Supplementary Figure S2A). The sensitivities of Δ*lexA*, Δ*lexB*, Δ*lexC*, Δ*lexABC*, and Δ*lexR* strains were also demonstrated by a filter disk assay for myxin-dependent growth inhibition. The inhibition circles of myxin against the above mentioned mutants increased with the growing concentrations of myxin (0.125, 0.25, 0.5 μg), while 0.5 μg of myxin had little effect on the growth of the wild type (Supplementary Figure S3). These results demonstrated the important roles of the RND system LexABC and the regulator LexR in self-resistance of *L. antibioticus* OH13 against myxin.

**TABLE 1:** Susceptibility of *L. antibioticus* OH13 and its derivatives to different concentrations of myxin.

**Growth status of *L. antibioticus* strains**												
**Myxin (μg/mL)**	**24 h**						**48 h**					
	**OH13**	**△*lexA***	**△*lexB***	**△*lexC***	**△*lexABC***	**△*lexR***	**OH13**	***△lexA***	***△lexB***	***△lexC***	***△lexABC***	***△lexR***
0	++++	++++	++++	++++	++++	++++	+++++	+++++	+++++	+++++	+++++	+++++
2	++++	+++	+++	+++	+++	+++	+++++	+++++	+++++	+++++	+++++	+++++
4	++++	++	++	++	++	++	+++++	++++	++++	++++	++++	++++
8	+++	+	+	+	+	+	+++++	+	+	+	+	+
16	+	−	−	−	−	−	++++	−	−	−	−	−

*Corresponding to Figure 2B. “****+****” indicated obvious colony grown, and different numbers of* “***+***” *showed various growth level. “***−***” indicated no colony grown.*

### LexABC System and LexR Are Required for Normal Myxin Production

**FIGURE 3:**
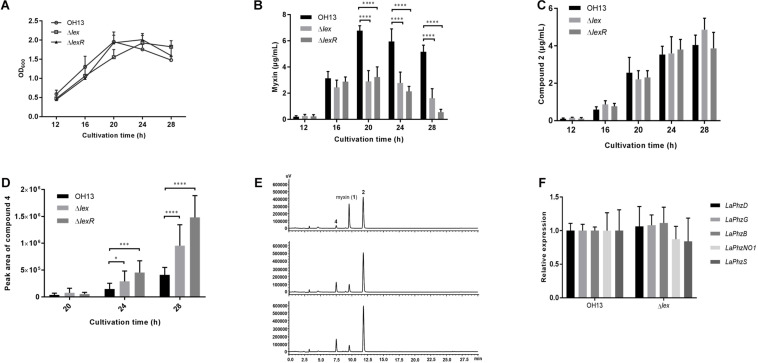
LexABC pump and LexR are required for normal myxin production of *L. antibioticus* OH13. **(A)** Growth curves of OH13, Δ*lex* and Δ*lexR* strains cultured in 1/10 TSB for 12, 16, 20, 24, and 28 h after inoculation. **(B)** Myxin production in OH13, Δ*lex* and Δ*lexR* strains grown in 1/10 TSB for different time points. Supernatant of cultures was extracted and detected for myxin yield. **(C)** Compound **2** production in OH13, Δ*lex* and Δ*lexR* strains grown in 1/10 TSB for different time points. **(D)** Compound **4** production in OH13 (top), Δ*lex* (middle) and Δ*lexR* (bottom) strains grown in 1/10 TSB for different time points. The production of compound **4** was indicated with peak area of HPLC. **(E)** HPLC analysis of extract from OH13, Δ*lex* and Δ*lexR* strains at cultivation time of 28 h. **(F)** Transcriptional expression of myxin biosynthetic genes in strain OH13 and *lex* mutant. Data shown in graph are the means from three independent experiments and error bars are standard deviations. Asterisks indicate statistically significant difference according to *t*-test in GraphPad Prism 7.0. ^∗^*p* < 0.05, ^∗∗∗^*p* < 0.001, ^****^p < 0.0001.

To investigate the role of the LexABC system and LexR in cell growth and myxin production, cell growth curves and myxin production of wild-type strain OH13, Δ*lexABC* (hereafter referring to *lexABC* as *lex*) and Δ*lexR* were compared in 1/10 TSB medium. At 12 h, the growth rate and myxin production were comparable between strain OH13 and the *lex* mutant (Figure 3A). The growth of the *lex* mutant was delayed at 16 and 20 h but caught up with OH13 at 24 h (Figure 3A). However, the accumulation of myxin in the *lex* mutant was flat since 16 h, which was over 2-fold less than that in OH13 (Figure 3B). Deletion of *lexR* did not affect the bacterial growth but resulted in similar decrease level of myxin production with *lex* mutant (Figures 3A,B). These results indicated that *L. antibioticus* could endure a certain concentration of myxin without the LexABC efflux pump or LexR. To explore whether myxin was converted to its derivatives in *lex* or *lexR* mutant, we detected the yield of other main phenazine compounds. The results showed that compound **2** (Figure 1) was not significantly changed in *lex* or *lexR* mutant compared with that in OH13 (Figure 3C). Meanwhile, there was an increased peak corresponding to phenazine compound **4** (Figure 1) drawing our attention (Figure 3E). Quantitative analysis revealed that compound **4** was significantly increased in *lex* or *lexR* mutant compared to OH13 at the cultivation time of 24 and 28 h (Figure 3D). Compounds **2** and **4** showed inhibitory activity against certain bacteria but not as strong as myxin in the tests we conducted (Zhao et al., 2016). The results suggested that *L. antibioticus* converted myxin to its derivatives to protect itself when the LexABC efflux pump or LexR was deficient. To further determine whether the missing of LexABC pump or LexR enhances the intracellular myxin accumulation, we analyzed the intracellular phenazines of OH13, Δ*lex* and Δ*lexR* strains. The result showed that compound **2** but not myxin accumulated in the intracellular, but it was not as expected that *lex* and *lexR* mutants had a higher phenazine accumulation than the wild type (Supplementary Figure S4).

To understand whether the absence of the LexABC efflux pump influences myxin biosynthesis at the transcriptional level, the relative gene expression of myxin biosynthetic genes were compared between OH13 and the *lex* mutant. The detected genes included three core genes, *LaPhzD*, *LaPhzG*, and *LaPhzB*, for the formation of the myxin precursor, and two modification genes, *LaPhzNO1*, encoding Baeyer-Villiger similar flavoproteins responsible for phenazine *N*-oxidation, and *LaPhzS*, encoding FAD-dependent enzyme for catalyzing decarboxylative hydroxylation of PDC (Zhao et al., 2016). The qPCR showed that the expression of these biosynthetic genes was not significantly changed in the absence of *lex* (Figure 3F). These results suggested that the lack of the LexABC efflux pump affected myxin production but not by impacting its biosynthesis at the transcriptional level. The LexABC efflux pump is necessary for protecting normal cell growth and myxin production under a high level of myxin accumulation.

### Expression of the LexABC Efflux Pump Is Induced by Myxin

**FIGURE 4:**
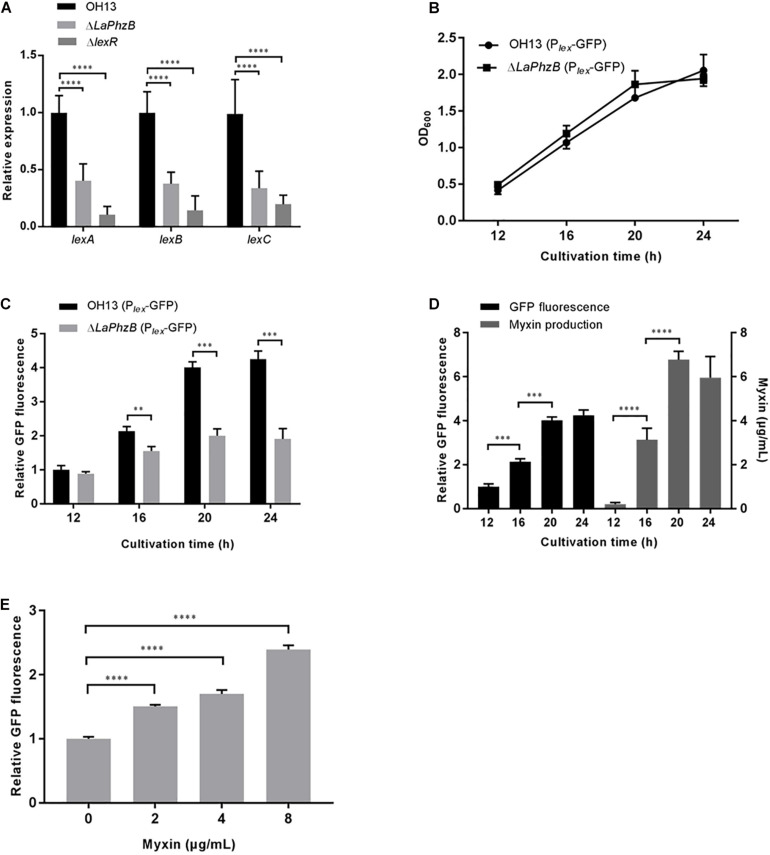
Expression of LexABC was stimulated by myxin. **(A)** qPCR analysis of *lexA*, *lexB*, *lexC* genes expression in strain OH13, Δ*LaPhzB*, Δ*lexR* strains. **(B)** Growth curves of strains OH13 (P*_lex_*-GFP) and Δ*LaPhzB* (P*_lex_*-GFP) cultured in 1/10 TSB for 12, 16, 20, and 24 h after inoculation. **(C)** Relative GFP fluorescence in strains OH13 (P*_lex_*-GFP) and Δ*LaPhzB* (P*_lex_*-GFP) at different time points. The fluorescence intensity in strain OH13 (P*_lex_*-GFP) at 12 h was normalized as 1. **(D)** Presentation of the relationship between myxin concentration and gene expression of *lexABC*. Data from Figures 3B,4C of strain OH13. **(E)** Relative GFP fluorescence in strain Δ*LaPhzB* (P*_lex_*-GFP) added with 0, 2, 4, 8 μg/mL myxin. The fluorescence intensity in strain Δ*LaPhzB* (P*_lex_*-GFP) without myxin was normalized as 1. Data shown in graph are the means from three replicates and error bars are standard deviations. Asterisks indicate statistically significant difference according to *t*-test in GraphPad Prism 7.0. ^∗∗^*p* < 0.01, ^∗∗∗^*p* < 0.001, ^****^p < 0.0001.

Our previous study showed that deletion of *LaPhzB* abolished production of all of the phenazines (Zhao et al., 2016). The transcriptional levels of the LexABC efflux system genes in strain OH13 and the *LaPhzB* mutant were determined by qPCR. The results indicated that the expression levels of *lexA*, *lexB*, and *lexC* were significantly down-regulated in the *LaPhzB* mutant compared to that in the wild type (Figure 4A). The decreasing expression level of *lex* in the myxin deficient mutant suggested that myxin possibly stimulated LexABC expression.

To test this assumption, we constructed strains OH13 (P*_lex_*-GFP) and Δ*LaPhzB* (P*_lex_*-GFP) that expressed GFP under the control of the *lex* promoter. The growth rate of both strains was about the same (Figure 4B), but the fluorescence of strain OH13 (P*_lex_*-GFP) exhibited a substantial increase along with bacterial growth from 12 to 20 h, whereas the fluorescence in strain Δ*LaPhzB* (P*_lex_*-GFP) was stable since the 16th h of cultivation. The relative fluorescence of Δ*LaPhzB* (P*_lex_*-GFP) was approximately 50% lower than that of OH13 (P*_lex_*-GFP) at 20 and 24 h (Figure 4C). To show the relationship between myxin concentration and gene expression of *lexABC*, the myxin production and GFP fluorescence of strain OH13 were combined in one diagram (data from Figures 3B, 4C). Expression of *lexABC* genes was consistent with myxin production during cell growth (Figure 4D), suggesting that *lexABC* expression is influenced by myxin. To further confirm these results, the expression of *lexABC* was analyzed in strain Δ*LaPhzB* (P*_lex_*-GFP) supplemented with different concentrations of myxin. The data showed that 2, 4, and 8 μg/mL of myxin enhanced the expression of LexABC by 1. 5-, 1. 7-, and 2.4-fold, respectively, compared with the control (Figure 4E). These results suggested that the expression of LexABC was induced by myxin and was concentration dependent.

### LexR Could Positively Regulate LexABC Efflux Expression

The regulation mechanism of how these transporters sense and respond to the accumulation of myxin remains unclear. As described above, deletion of *lexR* resulted in strains more sensitive to myxin and decreased myxin production, which was similar with the LexABC system deficient strains (Figures 2B, 3B). LexR was predicted as a LysR family transcriptional regulator, so the transcriptional level of *lexABC* was compared between the wild type and *lexR* mutant by qPCR. The gene expression level of *lexA*, *lexB*, and *lexC* was significantly reduced by more than 2-fold in the *lexR* mutant (Figure 4A). The results indicated that LexR could positively regulate the expression of LexABC system and play an essential role in myxin resistance.

### Phylogenetic Analysis of the LexABC Efflux Pump

**FIGURE 5:**
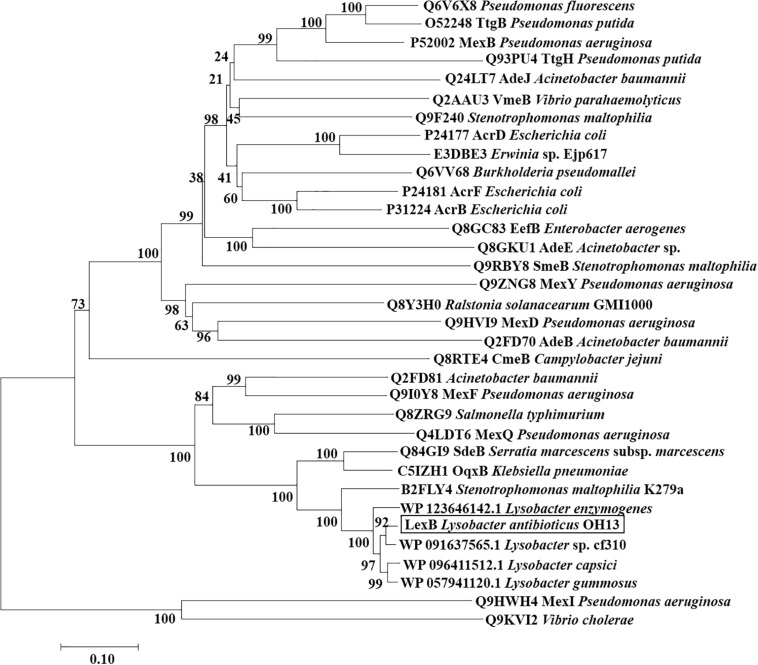
Phylogenetic tree of LexB and other RND proteins. Neighbor-joining tree based on the amino acid sequences of RND family proteins related to LexB was generated using MEGA 7.0 with 1,000 bootstrap replicates. The black box indicates the position of LexB from *L. antibioticus* OH13. The sequences of four *Lysobacter* species were from NCBI, and the other sequences were obtained from TCDB (Transporter Classification Database).

To elucidate the evolutionary relationship of the LexABC efflux with other RND efflux complexes, a phylogenetic tree was constructed with LexB and its homolog proteins (amino acid similarity above 53%), which were inner membrane proteins and functional in the recognition and binding of substrates. The phylogenetic tree contained 34 proteins from various bacterial species. LexB clustered together with the putative RND proteins from the other four *Lysobacter* strains in the tree, which showed that LexB homologs were conserved in *Lysobacter* species. The close homologs were the RND protein of *Stenotrophomonas maltophilia* K279a and SdeB from *Serratia marcescens* subsp. *marcescens*, which mainly exports fluoroquinolones (Kumar and Worobec, 2005; Chen et al., 2011). MexI from *P. aeruginosa*, which was characterized for *Pseudomonas* quinolone signal (PQS) (Aendekerk et al., 2005) and phenazine efflux (Sakhtaha et al., 2016), also showed close evolutionary relationship with LexB (Figure 5). The results indicated that the closely related RND homologs presented similarity in the substrates.

## Discussion

In this study, we identified a novel RND efflux pump, LexABC, which plays an important role in myxin resistance and production. LexABC confers resistance against phenazines such as myxin and PMS. We revealed that LexABC expression was sufficiently induced by myxin and potentially regulated by the LysR-type transcriptional regulator LexR.

The RND superfamily is common in Gram-negative bacteria and is a major contributor to multidrug resistance among human pathogens such as *Enterococcus faecium*, *Staphylococcus aureus*, *Klebsiella pneumoniae*, *Acinetobacter baumannii*, *P. aeruginosa*, and *Enterobacter* species (Li and Nikaido, 2004, 2009). Moreover, RND efflux pumps have also been reported in plant pathogens, for example, *Erwinia amylovora* (Pletzer and Weingart, 2014), *Dickeya species* (Liang et al., 2019), and *Pseudomonas syringae* (Stoitsova et al., 2008). Research into the RND efflux pump in biocontrol bacteria has been limited. Our study will contribute to the self-resistance of antibiotic-producing bacteria and provide new insight into pathogen multidrug resistance.

The results showed that the inactivation of any of the *lexABC* genes or all three genes could cause the same level of growth defect in presence of myxin. These findings demonstrated that the function of any component of LexABC is indispensable. The growth of the *lex* mutant lagged behind the wild type in the early stage and then caught up at a late stage in 1/10 TSB. The yield of myxin peaked at 20 h and then went down in the wild type, whereas the accumulation of compound **2** and **4** increased along with the increase of cultivation time (Figures 3B–D). The results suggested that when the accumulation of myxin reached a certain concentration which the strains can’t endure, myxin could be partly converted to its derivatives to protect bacteria from the toxicity. The production of myxin was significantly reduced in the *lex* and *lexR* mutant compared to the wild type after 20 h cultivation, and the yield of compound **2** didn’t show noteworthy change but compound **4** increased (Figures 3B–D). So the missing myxin in lex or lexR mutant was supposed to be converted into compound **4**. Compound **2** can be converted into compound **4** with *O*-methylation by *O*-methyltransferase LaPhzM (Jiang et al., 2018), but how myxin is transformed into compound **2** still needs study. Myxin or its derivatives did not show an increasing accumulation in the intracellular of *lex* and *lexR* mutant compared to the wild type (Supplementary Figure S4). Moreover, *lex* mutant had comparative myxin biosynthetic genes expression level to OH13 (Figure 3F). These results indicated that *L. antibioticus* protected itself by reducing myxin production when the LexABC system was disrupt but without affecting the biosynthesis of this compound. Antibiotic-producing bacteria usually involved several resistance strategies to protect themselves from suicide, and the LexABC efflux pump may be involved in the first line of defense in *L. antibioticus*. With the loss of LexABC efflux pump, the conversion of myxin to its derivatives was another defense line. The gene *LaPhzX* encoding putative small monooxygenase in myxin biosynthetic gene cluster, which is similar to ActVA-Orf6, TcmH (Zhao et al., 2016) and PumA (Sporer et al., 2018), has not been characterized. PumA was reported to contribute to phenazine resistance and was required for the conversion of PMS to unique phenazine metabolites, but its exact function was not clear (Sporer et al., 2018). We predict LaPhzX may contribute to the defense line of myxin, but further study is needed to determine its function in myxin resistance.

The expression level of the genes *lexABC* were increased along with the accumulation of myxin, and deletion of the myxin biosynthetic gene *LaPhzB* caused a decrease of the transcriptional expression of *lexABC* (Figures 4A,D). Moreover, exogenous addition of 8 μg/mL myxin to the *LaPhzB* mutant could enhance *lexABC* expression by more than 2-fold compared with that of the control (Figure 4E). These results demonstrated that myxin was not only an antibiotic but also physiologically an elicitor of the LexABC RND pump. This is similar with the famous phenazine PYO and its intermediate 5-methylphenazine-1-carboxylic acid (5-Me-PCA) which induce expression of the RND efflux pump-encoding operon *mexGHI-opmD*. MexGHI-OpmD transporter confers resistance to 5-Me-PCA, and its structurally similar, synthetic phenazine PMS (Sakhtaha et al., 2016). Disruption of LexABC system also led to *L. antibioticus* strains arrest of growth in presence of 50 and 100 μg/mL of PMS (Supplementary Figure S2B). The phylogenetic tree showed that the LexB RND protein was close to MexI. LexA, LexB, and LexC showed similarity to MexH, MexI and OpmD, respectively, but there was no additional MexG homolog adjacent which is an anomalous cytoplasmic membrane component not required for the normal export of 5-Me-PCA and PMS. The induction of *mexGHI-opmD* by phenazine was mediated by activating the redox-active transcription factor SoxR (Dietrich et al., 2006; Sakhtaha et al., 2016). SoxR acts as a redox sensor and is activated by redox-active compounds through oxidation or nitrosylation of its [2Fe-2S] cluster (Singh et al., 2013). Phenazine/SoxR/MexGHI-OpmD system is a model for regulation of natural product efflux and self-protection in Gram-negative antibiotics producing bacteria. We hypothesize a specific pathway to sense myxin production in self-protection against the detrimental effect of this antibiotic. The gene *lexR* encodes LysR family transcriptional regulator, which is located upstream of the *lexABC* locus, was studied. Deletion of this gene resulted in growth defect of *L. antibioticus* in presence of myxin and decreased myxin production (Figures 2, 3B). Moreover, the gene expression of *lexA*, *B*, *C* was down-regulated significantly in *lexR* mutant (Figure 4A). Our data showed that LexR played an important role in myxin resistance by positively regulating *lexABC* expression. Besides SoxR, MexT also functions as a redox-responsive regulator inducing the MexEF-OprN efflux system (Fargier et al., 2012). Myxin is a redox-cycling compound with *N*-oxidation, however, LexR is not similar to MexT or SoxR. A different signaling mechanism might underlying the regulatory linkage between LexR and LexABC pump. Though myxin/LexABC system shares much similarity with phenazine (5-Me-PCA and PMS)/MexGHI-OpmD system, myxin is featured as *N*-oxidation and *O*-methylation, which is different from 5-Me-PCA and PMS of *N*-methylation. Therefore, the LexABC-based myxin resistance mechanism and its regulatory network potentially will be a novel self-resistance model in antibiotic producing bacteria.

In conclusion, we report resistance mechanisms against myxin, which is a heterocyclic *N*-oxides penazine with a potent broad spectrum of antimicrobial activities. The LexABC efflux pump confers myxin resistance, and myxin conversion played more important role in the resistance mechanisms after LexABC loss. The Lex RND efflux pump is potentially regulated by a LysR family regulator LexR. Significantly, this study also revealed a signaling role of myxin in the induction of *lexABC* expression at the transcriptional level, which further extended our perceptions about myxin.

## Materials and Methods

### Bacterial Strains, Growth Conditions, and General DNA Manipulations

The strains and plasmids used in this study are listed in Table 2. *L. antibioticus* OH13 and derivatives were routinely grown at 28°C in nutrient broth (NB, 3 g beef extract, 1 g yeast extract, 5 g tryptone, 10 g sucrose, pH 7.0–7.2, in one liter distilled water; without sucrose, NBWS), nutrient agar (NA, 1 L NB with 17 g agar; without sucrose, NAWS), 1/10 tryptic soy broth (TSB). 1/10 TSB medium is more appropriate for myxin production. *E. coli* strains were routinely grown at 37°C in Luria-Bertani (LB) broth. Where necessary, media were supplemented with gentamycin (25 or 50 μg/mL). Plasmid preparation and DNA extraction were carried out with Omega kits (Norcross, GA).

**TABLE 2:** Strains and plasmids used in this study.

**Strains or plasmids**	**Description**	**Sources or references**
**Strains**		
***Lysobacter antibioticus***		
OH13	Wild-type strain	Zhao et al., 2016
OH13(P*_lex_*-GFP)	OH13 with *lex* promoter driving *gfp* expression	This study
△*LaPhzB*	OH13 with deletion in *LaPhzB*	Zhao et al., 2016
△*LaPhzB*(P*_lex_*-GFP)	△*LaPhzB* with *lex* promoter driving *gfp* expression	This study
△*lexA*	OH13 with deletion in *lexA*	This study
△*lexB*	OH13 with deletion in *lexB*	This study
△*lexC*	OH13 with deletion in *lexC*	This study
△*lexABC*	OH13 with deletion in *lexABC*	This study
△*lexR*	OH13 with deletion in *lexR*	This study
△l*exA*(pBBR)	△*lexA* carrying pBBR1-MCS5 vector, Gm^r^	This study
△*lexA*(pBBR-*lexA*)	△*lexA* carrying pBBR-*lexA* vector, Gm^r^	This study
△*lexB*(pBBR)	△*lexB* carrying pBBR1-MCS5 vector, Gm^r^	This study
△*lexB*(pBBR-*lexB*)	△*lexB* carrying pBBR-*lexB* vector, Gm^r^	This study
△*lexABC*(pBBR)	△*lexABC* carrying pBBR1-MCS5 vector, Gm^r^	This study
△*lexABC*(pBBR-*lexABC*)	△*lexABC* carrying pBBR-*lexABC* vector, Gm^r^	This study
***Escherichia coli***		
DH5α	F- φ80dlacZΔM15 Δ(lacZYA-argF)U169 deoR recA1 endA1 hsdR17(r_k_-,m_k_ +) phoA supE44 λ-thi-1 gyrA96	TransGen, China
**Plasmids**		
pJQ200SK	Suicide cloning vector, Gm^r^	Quandt and Hynes, 1993
pJQ200SK-*lexA*	pJQ200SK with two flanking fragments of *lexA*	This study
pJQ200SK-*lexB*	pJQ200SK with two flanking fragments of *lexB*	This study
pJQ200SK-*lexC*	pJQ200SK with two flanking fragments of *lexC*	This study
pJQ200SK-*lexABC*	pJQ200SK with two flanking fragments of *lexABC*	This study
pJQ200SK-*lexR*	pJQ200SK with two flanking fragments of *lexR*	This study
pJQ200SK-P*_lex_-gfp*	pJQ200SK with two flanking fragments of *lexA* start site and *gfp*	This study
pBBR1-MCS5	Broad host range cloning vector, lacZ, Gm^r^	Kovach et al., 1995
pBBR-*lexA*	pBBR1-MCS5 carrying ORF of *lexA* and its promoter, Gm^r^	This study
pBBR-*lexB*	pBBR1-MCS5 carrying ORF of *lexB* and its promoter, Gm^r^	This study
pBBR-*lexABC*	pBBR1-MCS5 carrying ORF of *lexABC* and its promoter, Gm^r^	This study

### Gene Deletion and Complementation

The oligonucleotide primers used in this study are listed in Supplementary Table S1. DNA manipulation was conducted by following the methods described previously (Zhao et al., 2016). Briefly, for gene in-frame deletions, fragments containing the upstream and downstream regions of the target genes were cloned into pJQ200SK (Quandt and Hynes, 1993) and transformed into *E. coli* DH5α for the construction of gene in-frame deletion constructs. The final constructs were transformed into the competent cells of OH13 wild type or its derivatives by electroporation. The gentamycin-resistant colonies were screened by PCR using primers to identify the single crossover mutants. Then, the single crossover mutants were grown on NAWS medium containing 8% sucrose, which would screen for deletion mutants through a second homologous recombination of the flanking regions. The mutants were verified by PCR using primers.

For complementation, the open reading frames (ORFs) of the target genes with their own promoters were cloned into pBBR1-MCS5 (Kovach et al., 1995). The expression constructs were confirmed by PCR and DNA sequencing and introduced into wild type or corresponding mutants by electroporation. The complementation strains were screened on NAWS medium containing gentamycin and were validated by PCR.

### Preparation and Analysis of Myxin

*L. antibioticus* OH13 was cultivated for 24 h in a 250 mL flask containing 50 mL NB medium. The seed culture was then transferred into 200 mL 1/10 TSB medium in a 1 liter flask (a total of 50 flasks). The flasks were placed in incubators for 24 h with shaking at 180 rpm at 28°C. Then, the cultures were extracted with equal volumes of ethyl acetate, and the organic phase was evaporated to dryness under vacuum to yield the crude extract. The crude extract was chromatographed over a Sephadex LH-20 column using 1:1 CH_2_Cl_2_-MeOH as the elution solvent. The fractions were further purified by reversed-phase high-performance liquid chromatography (HPLC) with CH_3_CN in H_2_O (Agilent-C18, 5 μm, 9.4 × 250 mm) under 283 nm with a 4.0 mL/min flow rate.

To analyze myxin production in OH13 and its derivatives, equal volume of ethyl acetate was used to extract 1 mL supernatant of cultures grown in 1/10 TSB. The crude extract was transferred to an Eppendorf tube and fully evaporated. Then, the residues were dissolved with 200 μL methanol and prepared for HPLC analysis. To determine the production of intracellular myxin, 1 mL culture grown for 20 h in 1/10 TSB was harvested and centrifuged at 12,000 rpm for 1 min. Supernatant was removed and the pellet was washed with 1/10 TSB for twice. The pellets were extracted with 1 mL of ethyl acetate overnight. Then the extract was dissolved in methanol and subjected to HPLC.

The detection of phenazines was through HPLC (Agilent SB-C18, 250 × 4.6 mm) with a 1.0 mL/min flow rate. The mobile phase was 20–45% CH_3_CN in H_2_O from 0 to 5 min, 45 to 50% CH_3_CN in H_2_O from 5 to 19 min, 50 to 60% CH_3_CN in H_2_O from 19 to 20 min, 60 to 100% CH_3_CN in H_2_O from 20 to 23 min, 100% CH_3_CN from 23 to 27 min, 100 to 20% CH_3_CN in H_2_O from 27 to 28 min, and 20% CH_3_CN in H_2_O from 28 to 30 min.

### Susceptibility Test of *L. antibioticus* to Phenazines

The susceptibility of the OH13 wild type and mutants to phenazines was determined by agar screening tests. Myxin was dissolved in DMSO, whereas PMS was dissolved in water. Liquid precultures of each strain to be tested were grown in NB medium to 1.0 of OD_600_. Five microliters of cultures were spotted on NA media containing the indicated concentrations of phenazines and incubated at 28°C. After 24 and 48 h of growth, the colonies were imaged using a Canon camera. For the inhibition assays of myxin against *Lysobacter* strains, bacterial suspensions with OD_600_ 1.0 were 100-fold diluted and then spread on NA plates, and subsequently different concentrations of myxin were dropped on filter papers.

### RNA Isolation and qRT-PCR

Bacterial cells were cultured and harvested at an OD_600_ of approximately 1.0. RNA extraction was performed using the Omega bacterial RNA extraction kit (Omega biotek, Norcross, GA) by following the manufacturer’s instructions. The concentration of RNA, *A*260/*A*280 and *A*260/*A*230 ratios were determined by using an Eppendorf BioPhotometer plus. In qPCR analysis, a 250 ng RNA sample was used for genomic DNA elimination and cDNA synthesis by a Vazyme HiScript II Q RT SuperMix (with gDNA wiper) by following the manufacturer’s protocol. Specific primers for qRT-PCR (Supplementary Table S1) were designed by Primer 3 online. The housekeeping gene 16S rDNA was used as a reference. The qPCR analysis was conducted on a Quantstudio 6 Flex system (Applied Biosystems) using ChamQ^TM^ SYBR qPCR Master Mix (Vazyme) with the following cycle profile: 1 cycle at 95°C for 30 s, followed by 40 cycles at 95°C for 5 s, 60°C for 34 s, and 1 cycle at 95°C for 30 s, 95°C 60 s, and 95°C for 15 s. The experiment was repeated three times, each time with triplicates. Data were analyzed using the 2^–Δ^ ^Δ^ ^CT^ method.

### Construction of GFP Expressing Strains and Flow Cytometry Analysis

Strains expressing GFP under the control of P*_lex_* were generated by homologous recombination. The fragment of *gfp* was integrated into the chromosome of *L. antibioticus* located in front of the *lexA* start site. We amplified regions ∼600 bp up and downstream of the *gfp* insertion locus using OH13 genomic DNA as a template, whereas *gfp* was amplified from a synthetic plasmid. The fragments were cloned into pJQ200SK using homologous recombination with a *pEASY*^®^ -Uni Seamless Cloning and Assembly Kit (TransGen, China). The construct was transferred into the OH13 wild type and *LaPhzB* mutant strains through electroporation. After twice homologous recombination, the *gfp* fragment was integrated into the chromosome of *L. antibioticus*, subsequently obtaining the OH13 (P*_lex_*-GFP) and Δ*LaPhzB* (P*_lex_*-GFP) strains. For quantification of *lex* expression, strains were grown in 1/10 TSB medium for different time periods. The Δ*LaPhzB* (P*_lex_*-GFP) strain was also cultured in 1/10 TSB medium and supplied with different concentrations of myxin when the OD_600_ reached 0.6. GFP fluorescence was analyzed by detecting the mean fluorescence intensity (MFI) of 10,000 cells with a CytoFLEX flow cytometer (BD FACSJazz).

### Construction of the Phylogenic Tree

The amino acid sequence of LexB was applied to blast research in the Transporter Classification Database (TCDB)^1^ and NCBI. 29 sequences from TCDB and 4 sequences of *Lysobacter* species from NCBI were downloaded. The amino acid sequences of the RND family proteins related to LexB were aligned, and a neighbor-joining tree was constructed with MEGA 7.0 (Kumar et al., 2016).

## Data Availability Statement

The datasets presented in this study can be found in online repositories. The names of the repository/repositories and accession number(s) can be found in the article/Supplementary Material.

## Author Contributions

YZ contributed to conceptualization, investigation, writing and editing the manuscript, and funding acquisition. JL, TJ, and RH did the experiments and analyzed data. GX and HX modified the main body of the manuscript. FL contributed to review the manuscript, supervision, and funding acquisition. All authors contributed to the article and approved the submitted version.

## Conflict of Interest

The authors declare that the research was conducted in the absence of any commercial or financial relationships that could be construed as a potential conflict of interest.

## Footnotes

1 http://www.tcdb.org/
