# Supplementary material for: Resistance-Nodulation-Division Efflux Pump, LexABC, Contributes to Self-Resistance of the Phenazine Di-N-Oxide Natural Product Myxin in Lysobacter antibioticus
Source: Front Microbiol. 2021 Feb 17;12:618513. doi: 10.3389/fmicb.2021.618513 (PMC7927275; doi:10.3389/fmicb.2021.618513)
Supplement: Supplementary file 1 [file Image_1.pdf]

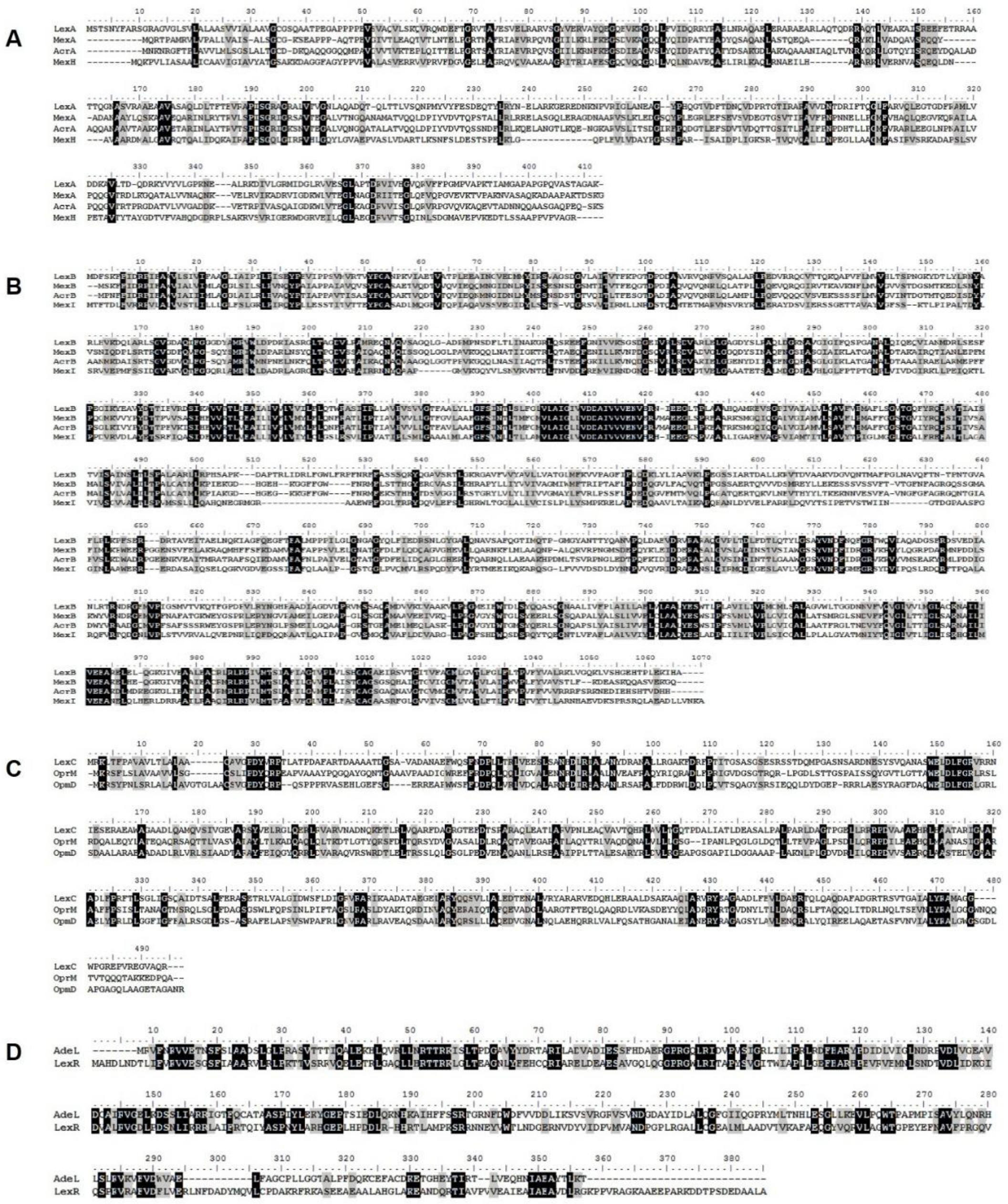

FIGURE S1 Multiple alignment of the amino acid sequence of LexA, LexB, LexC and LexR. **(A)** LexA from *L. antibioticus* OH13 (MW029615), MexA from *Pseudomonas aeruginosa* PAO1 (NP_249116.1), AcrA from *Escherichia coli* str. K-12 substr. MG1655 (NP_414996.1), MexH from *P. aeruginosa* PAO1 (NP_252895.1). **(B)** LexB from *L. antibioticus* OH13 (MW029616), MexB from *Pseudomonas aeruginosa* PAO1 (NP_249117.1), AcrB from *Escherichia coli* str. K-12 substr. MG1655 (NP_414995.1), MexI from *P. aeruginosa* PAO1 (NP_252896.1). **(C)** LexC from *L. antibioticus* OH13 (MW029617), OprM from *P. aeruginosa* PAO1 (NP_249118.1), OpmD from *P. aeruginosa* PAO1 (NP_252897.1). **(D)** LexR from *L. antibioticus* OH13 (MW029618), AdeL from *Acinetobacter baumannii* (AXV51761.1).
