# Supplementary material for: Resistance-Nodulation-Division Efflux Pump, LexABC, Contributes to Self-Resistance of the Phenazine Di-N-Oxide Natural Product Myxin in Lysobacter antibioticus
Source: Front Microbiol. 2021 Feb 17;12:618513. doi: 10.3389/fmicb.2021.618513 (PMC7927275; doi:10.3389/fmicb.2021.618513)
Supplement: Supplementary file 2 [file Image_2.pdf]

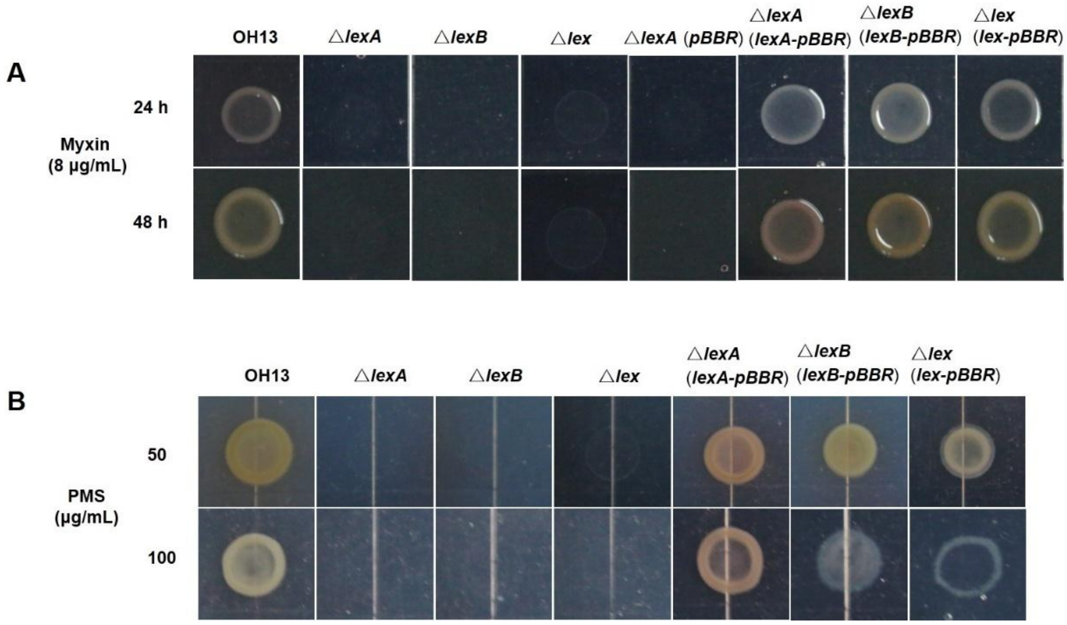

FIGURE S2 Growth of *L. antibioticus* OH13 and its derivatives on media containing myxin and PMS. **(A)** Strains cultured on media containing 8 μg/mL myxin for 24 and 48 h. **(B)** Strains cultured on media containing 50 and 100 μg/mL PMS for 48 h.
