# Supplementary material for: Resistance-Nodulation-Division Efflux Pump, LexABC, Contributes to Self-Resistance of the Phenazine Di-N-Oxide Natural Product Myxin in Lysobacter antibioticus
Source: Front Microbiol. 2021 Feb 17;12:618513. doi: 10.3389/fmicb.2021.618513 (PMC7927275; doi:10.3389/fmicb.2021.618513)
Supplement: Supplementary file 3 [file Image_3.pdf]

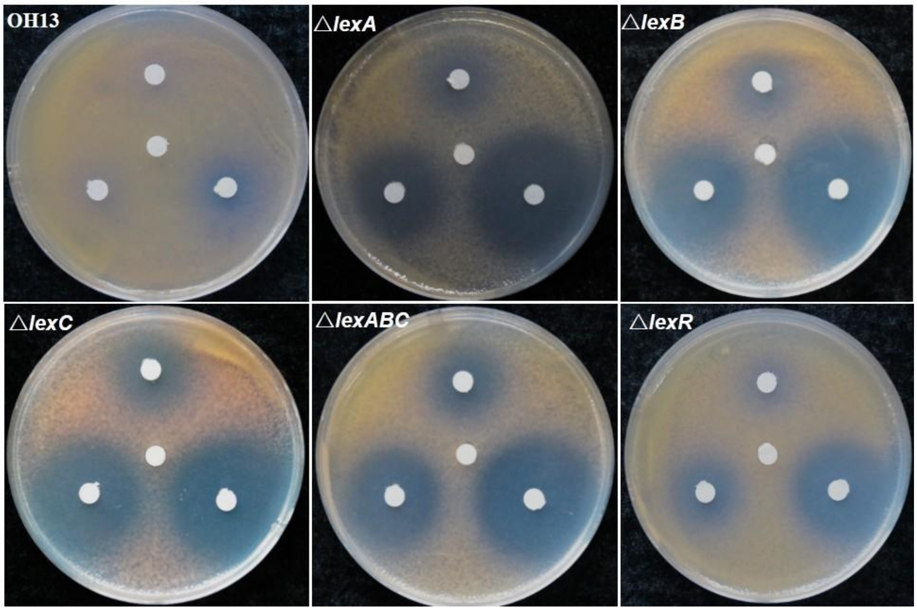

FIGURE S3 Inhibition assay of myxin against *L. antibioticus* OH13, *lexA*, *lexB*, *lexC*, *lexABC*, *lexR* mutants. On a plate, center, negative control; top, 0.125 μg myxin; left, 0.25 μg myxin; right, 0.5 μg myxin.
