# Supplementary material for: Resistance-Nodulation-Division Efflux Pump, LexABC, Contributes to Self-Resistance of the Phenazine Di-N-Oxide Natural Product Myxin in Lysobacter antibioticus
Source: Front Microbiol. 2021 Feb 17;12:618513. doi: 10.3389/fmicb.2021.618513 (PMC7927275; doi:10.3389/fmicb.2021.618513)
Supplement: Supplementary file 4 [file Image_4.pdf]

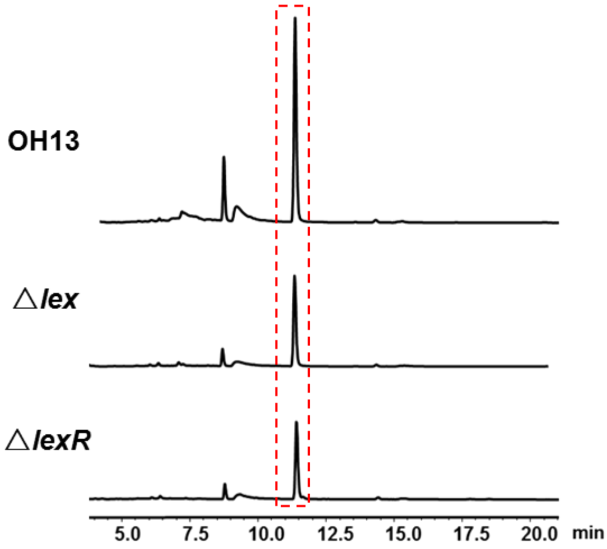

FIGURE S4 HPLC analysis of intracellular phenazine from OH13, Δ*lex* and Δ*lexR* strains. Dashed box indicates the compound **2**.
