# Supplementary material for: Resistance-Nodulation-Division Efflux Pump, LexABC, Contributes to Self-Resistance of the Phenazine Di-N-Oxide Natural Product Myxin in Lysobacter antibioticus
Source: Front Microbiol. 2021 Feb 17;12:618513. doi: 10.3389/fmicb.2021.618513 (PMC7927275; doi:10.3389/fmicb.2021.618513)
Supplement: Supplementary file 5 [file Table_1.docx]

| **TABLE S1** Primers used in this study |
| --- |
| Name Primer sequence (5′→3′) Usage |
| **Primers for in-frame gene deletion** |
| *lex*A-F1 GCTCTAGACATCGGATGCGCAACCTTGG  *lex*A-R1 GGAATTCCTGGCCCAGCGTTGATGGTTC *lexA* deletion  *lex*A-F2 GGAATTCCTACGCATGGATCTTCTCCAG  *lex*A-R2 CGGGATCCATGGACCGACCTGAGCTACC |
| *lexB*-F1 CGGGATCCCGAGGGTTTCCTTCTGGTTGTC  *lexB*-R1 GGAATTCCGGAGAAGATCCATGCGTAAG *lexB* deletion  *lexB*-F2 GGAATTCCCATGACGGATTCCTTGGTGC  *lexB*-R2 GCTCTAGAGCCGGTCGACTTCACCGACAAC |
| *lexC*-F1 GCTCTAGAGCTGCACCACCATCAGGAACAC  *lexC*-R1 GGAATTCCAAATAACCGCGGCAGCGTTC *lexC* deletion  *lexC*-F2 GGAATTCCGTAGTTGCTGGTGCTCATCG  *lexC*-R2 CGGGATCCCGAGTTCCTGCGCCGCAAGCTC |
| *lex*ABC-F1 GCTCTAGACATCGGATGCGCAACCTTGG  *lex*ABC-R1 GGAATTCCTGGCCCAGCGTTGATGGTTC *lexABC* deletion  *lex*ABC-F2 GGAATTCCGTAGTTGCTGGTGCTCATCG  *lex*ABC-R2 CGGGATCCCGAGTTCCTGCGCCGCAAGCTC |
| *lex*R-F1 CGGGATCCCGTCCCATGACGAGTGTCATTC  *lex*R-R1 GGAATTCCTGGGCAATGGCCTCCGTAAG *lexR* deletion  *lex*R-F2 GGAATTCCCCGGATAGGCAGCATCGTAG  *lex*R-R2 GCTCTAGAGCTTGCGTGGCGGTTAGGTGTG |
| **Primers for gene complementation** |
| *lexA*-HF CGGGATCCACCACGACGCCGACAGCGAA  *lexA* complementation  *lexA*-HR GCTCTAGAACGCTGCCGCGGTTATTTCG |
| pro-F cagcccgggggatccACCACGACGCCGACAGC promoter for *lexB* complementation  pro-R tttggaaaagtccatCGGGGGGTGGGTTCCT  *lexB*-HF ggaacccaccccccgATGGACTTTTCCAAATTTTTCATCGATC *lexB* complementation  *lexB*-HR ggcggccgctctagaGGATCTTCTCCAGAGGAGTGT |
| *lexA-*OF cagcccgggggatccACCACGACGCCGACAGC *lexABC* complementation  *lexA-*OR cgaacgctgccgcggTTATTTCGCCCCGGCCGT  *lexB-*OF gccggggcgaaataaCCGCGGCAGCGTTCGAA  *lexB-*OR ggtcaacttacgcatGGATCTTCTCCAGAGGAGTGT  *lexC-*OF ctctggagaagatccATGCGTAAGTTGACCTTTCCCG  *lexC-*OR ggcggccgctctagaTCAACGCTGGGCCACGC |

| **Primers for construction of pJQ200SK-P*_lex_*-*gfp*** |
| --- |
| P-lex-F1 tcctgcagcccggggAAGCTCGACGAGGATCTCCT two flanking of *lexA* start site  P-lex-R1 ttcacctttactcatCGGGGGGTGGGTTCCT  P-lex-F2 gaactctacaaatgaATGAGCACCAGCAACTACTTCG  P-lex-R2 tggcggccgctctagGAAACCAGCGTGGTCAGCT  gfp-F ggaacccaccccccgATGAGTAAAGGTGAAGAACTGTTCAC *gfp* fragment  gfp-R gttgctggtgctcatTCATTTGTAGAGTTCATCCATGCC |
| **Primers for qRT-PCR** |
| 16S-qF ACGGTCGCAAGACTGAAACT target 16S rDNA gene  16s-qR AAGGCACCAATCCATCTCTG |
| *LaPhzD*-qF CCCAAGATCCAGCCTTATGC target *LaPhzD* gene  *LaPhzD*-qR ATCGAAGCGGCATTCTTGAC |
| *LaPhzG*-qF ACCAGGCCATCGACAATCGT target *LaPhzG* gene  *LaPhzG*-qR CGCGGCCTTTCTGGCTTTTC |
| *LaPhzB*-qF GTGGTCGAGGACTATATGAG target *LaPhzB* gene  *LaPhzB*-qF GAAGATCTGCACGTTCTTCC |
| *LaPhzNO1*-qF CCCGCAGATCCCCGGGCGCGACA target *LaPhzNO1* gene  *LaPhzNO1*-qR CGGCTGCCGGCGGGCACGCAGAA |
| *LaPhzS*-qF ATCAACTGGGTCTGCATGGT target *LaPhzS* gene  *LaPhzS*-qR GGTACTCGAGGATCTGGGTG |
| *lexA-*qF AGCAACACGCTCTGCTGATA target *lexA* gene  *lexA-*qR GACTGGTCGTTCCTCGACAT |
| *lexB-*qF CAGGTAGGTCTGCAGGGTGT target *lexB* gene  *lexB-*qR GCGGGCTATCAGTTGTTCAT |
| *lexC-*qF GACCTGGTTGTCGGTGAAGT target *lexC* gene  *lexC-*qR AGCAGACCTACCTGCGCTAC |
